# Two patients with atypical low triiodothyronine syndrome: primary deiodinase abnormalities?

**DOI:** 10.1530/EDM-13-0055

**Published:** 2014-02-01

**Authors:** Gerald J M Tevaarwerk

**Affiliations:** 1 University of Western OntarioLondon, Ontario N6A 5C1Canada

## Abstract

Low triiodothyronine (T_3_) concentrations in the presence of normal thyroxine (T_4_) and TSH levels, referred to as the low T_3_ syndrome (LT_3_S), are common. LT_3_S may be caused by starvation, various non-thyroidal illnesses (NTIs) and some medications. Reverse T_3_ (rT_3_) concentrations are elevated in the more severely ill, and they characteristically fail to respond to exogenous levothyroxine (l-T_4_) therapy. The biochemical abnormalities have been explained on the basis of altered peripheral deiodinase activities. Herein, we report on two patients with hypothyroid symptoms who on testing were found to have LT_3_S. They were atypical clinically in not having LT_3_S due to any of the usual causes, had no increased rT_3_ concentrations, and had a normal negative TSH feedback response to l-T_4_. One (patient 1) had previously been diagnosed with Hashimoto's autoimmune primary hypothyroidism and was on l-T_4_ therapy. Both had T_4_ concentrations in the reference range. TSH levels were elevated in patient 1 and in the reference range in patient 2. Starting or increasing l-T_4_ doses resulted in no clinical improvement and no increase in T_3_ levels in spite of a marked increase in T_4_ levels. It is suggested that in the absence of the usual causes, lack of elevated rT_3_ levels, response to treatment and intact negative TSH feedback these two patients differ from the usual secondary causes of decreases in deiodinase activity. It is speculated that they may represent primary alterations in deiodinase enzymes possibly due to genetic variations in the deiodinase-encoding genes.

**Learning points:**

- LT_3_S is commonly found secondary to starvation, NTIs and use of some medications.
- Low T_3_ levels are the result of alterations in the activity of deiodinase enzymes.
- LT_3_S without the usual causes may represent a primary disturbance in deiodinase activity.

## Background

In normal, euthyroid individuals, 20% of the circulating active thyroid hormone triiodothyronine (T_3_) comes directly from the thyroid gland and 80% is produced in non-thyroidal tissues by the activation of the inactive prohormone thyroxine (tetraiodothyronine, T_4_) (1) (2). Low T_3_ concentrations are found during starvation and in various acute and chronic illnesses (3) (4) (5). This condition is variously referred to as the euthyroid sick syndrome (ESS), non-thyroidal illness (NTI) syndrome or the low T_3_ syndrome (LT_3_S) (4) (5) (6). In critically ill patients, T_4_ concentrations may also be low, and the degree of disturbance in the circulating thyroid hormone concentrations is related to the severity of the NTI, with a decrease in the T_3_ concentration only being more benign (5) (6). LT_3_S as found in ESS is caused by a decrease in enzymatic deiodination at the 5′ position of the outer, phenol ring of the iodothyronines (2) (7). In more severe NTIs, there is a further reduction in T_3_ concentrations due to increased deiodination at the 5 position of the inner, tyrosyl ring reducing it to the inactive diiodothyronine (T_2_) and producing inactive reverse T_3_ (rT_3_) from T_4_, thereby yet further reducing T_3_ generation because of decreased substrate availability (2) (7) (8). Evidence of a disturbance in the thyroid-stimulating hormone (TSH) feedback loop has also been found (4) (5) (6) (8). LT_3_S is also found in response to some drugs, including propylthiouracil (PTU) (9), beta-blockers (10) and amiodarone (11) (12).

The enzymes responsible for these alterations are the deiodinases (D) D1, D2 and D3 (2) (7). D2 is an activating enzyme that converts T_4_ to T_3_ (and rT_3_ to T_2_) and is found in the endothelial reticulum of skeletal muscle, CNS, pituitary, thyroid, heart muscle and brown adipose tissue. D3 is a deactivating enzyme that converts T_4_ to rT_3_ and T_3_ to T_2_ and is found in the CNS and other tissues. D1 has both activating and deactivating activities and is found in the plasma membranes of liver, kidney, thyroid and pituitary tissue. Together, theses three enzymes play a vital role in the maintenance of tissue and circulating thyroid hormone levels (2) (7).

The 5′-deiodinase activity of D1 is inhibited by fasting, PTU and amiodarone (3) (9) (11) (12). Melmed *et al*. (11) have shown that the decreased generation of T_3_ caused by chronic amiodarone administration in euthyroid patients causes a compensatory increase in TSH and T_4_ concentrations. It is accompanied by a more modest increase in rT_3_ concentrations, which is partially explainable by the mass action effect of the marked increase in TSH-stimulated T_4_ secretion by the thyroid gland, but in addition represents some increase in D1 activity through its increased half-life caused by the increased secretion of T_3_ by the intact thyroid gland in response to the increased TSH concentration (2) (7).

Treatment of LT_3_S secondary to NTIs with levothyroxine (l-T_4_) has generally been found to be unsuccessful (4) (5) (6).

Herein, we report on two patients with clinical evidence of hypothyroidism and LT_3_S without any of the usual causes.

## Case presentations

### Case 1

A 63-year-old man, diagnosed at the age of 31 years with primary hypothyroidism due to Hashimoto's autoimmune thyroiditis when he received the first of two mitral valve replacements, was referred for continued severe cold intolerance, low physical energy and marked mental lethargy in spite of free T_4_ concentrations being near the upper limit of the reference range on l-T_4_ replacement. His weight was 98 kg. There was no goitre. He was on warfarin and on candesartan, verapamil, furosemide and digoxin therapy for hypertension and congestive heart failure and on allopurinol therapy for gout. There was no history of herpes or any other chronic viral infection.

### Case 2

A 60-year-old woman was referred for the evaluation of fatigue, cold intolerance, muscle cramps, depression and obesity of long duration. Her weight was 99 kg. There was no goitre. She was on candesartan and nifedipine therapy for hypertension. There was no history of herpes or any other chronic viral infection.

## Investigation

### Case 1

Thyroid tests revealed elevated thyroid peroxidase antibody (TPOA) levels, free T_4_ levels near the upper limit of the reference range, markedly elevated TSH levels and low free T_3_ levels. The T_4_:T_3_ ratio was high, rT_3_ concentrations were in the normal reference range, and the T_4_:rT_3_ ratio was more than double mid-normal. Plasma iodine concentration was within the reference range (Table 1).

**Table 1:** Clinical features and thyroid hormone levels in the two patients with atypical low T_3_ syndrome

**Patient, age, gender and treatment**	**TSH** (mU/l)	**T_4_** (pmol/l)	**T_3_** (pmol/l)	**T_4_/T_3_** (Ratio)	**rT_3_** (nmol/l)	**T_4_/rT_3_** (Ratio)
1. 63 years, male and TTPH on 75 μg l-T_4_	**38.8**	19.2	**3.0**	**6.4**		
Repeat on 75 μg l-T_4_	**36.5**	17.5	**3.1**	**5.6**	0.19	**92.1**
On 100 μg l-T_4_	**20.6**	**23.6**	**3.4**	**6.9**		
Repeat on 100 μg l-T_4_	**14.7**	**22.5**	**3.1**	**7.3**	0.23	**97.8**
2. 60 years, female and before treatment	1.50	19.8	**3.4**	**5.8**	–	–
On 150 μg l-T_4_	**<** **0.06**	**21.6**	**3.3**	**6.5**	0.54	40.0
**Analytical units**						
Normal reference range	0.38–5.5	10.5–20	3.5–6.5		0.14–0.54	
Mid-normal values	1.5	15.3	5.0	3.1	0.34	45

TTPH, thyroxine-treated primary hypothyroidism. Analyte levels are given in bold if outside the reference range. Patient 1: plasma iodine: 0.29 (*n*: 0.24–0.63 μmol/l).

### Case 2

Thyroid tests revealed normal TPOA levels and TSH and T_4_ levels within their reference ranges, but low T_3_ concentrations and an elevated T_4_:T_3_ ratio (Table 1).

Thyroid hormone measurements were carried out by LifeLabs Laboratory Services, 3680 Gilmore Way, Burnaby, BC, V5G 4V8, Canada, using a competitive immunoassay with direct chemiluminescent technology for TSH, T_4_ and T_3_ and electrochemiluminescent immunoassay for TPOA. The rT_3_ concentration was measured by Associated Regional and University Pathologist (ARUP), Inc., 500 Chipeta Way, Salt Lake City, UT, 84108, USA, using a competitive RIA. Plasma iodine measurement was carried out by the London Laboratory Services Group, PO Box 5010, London, ON, N6A 5W9, Canada, using high-resolution inductively coupled plasma mass spectroscopy.

## Treatment

### Case 1

Increasing l-T_4_ dose to 100 μg had no effect on the symptoms in spite of T_4_ concentrations rising to supraphysiological levels, TSH concentrations falling to half their previous levels, a minimal to no increase in T_3_ concentrations, and a further rise in the T_4_:T_3_ ratio. The rT_3_ concentration rose minimally and continued to fall within the reference range, and the T_4_:rT_3_ ratio remained more than double normal (Table 1). Because of the patient's heart failure, it was thought inadvisable to further increase the dose of l-T_4_.

### Case 2

Treatment with 150 μg l-T_4_ had no clinical effect, increased T_4_ concentrations and T_4_:T_3_ ratio, and suppressed TSH concentrations, but had no effect on T_3_ concentrations. The rT_3_ concentration remained within the reference range, and the T_4_:rT_3_ ratio was near the mid-normal level (Table 1).

## Discussion

Herein, we report on two patients with atypical LT_3_S in that they reported none of the usual causes such as NTIs, including chronic viral infections, starvation and mediations known to cause impaired T_3_ generation or its increased destruction. Their rT_3_ concentrations were not elevated, as is the case in more severe NTIs or if on amiodarone therapy (2) (7) (8) (11) (12). In both patients, the negative TSH feedback mechanism was intact in that their TSH levels fell in response to increasing doses of l-T_4_, albeit that in patient 1 the levels remained markedly elevated. Both presented with clinical symptoms of hypothyroidism, but neither responded clinically to elevating the T_4_ concentrations to supraphysiological levels, and nor was there a consistent increase in T_3_ concentrations.

The findings in patient 1 are reminiscent of those found in euthyroid patients on amiodarone therapy as reported by Melmed *et al*. (11) and Rosene *et al*. (12), except that TSH levels remained markedly elevated even at supraphysiological concentrations of T_4_ and that the T_4_:rT_3_ ratio was elevated (Table 1). It is consistent with an absent response of the thyroid gland to elevated TSH levels because of the patient's primary hypothyroidism. In fact, he might not have come to attention if he had not also had primary hypothyroidism in that the elevated TSH levels would presumably have resulted in increased secretion of thyroid hormones and suppression of TSH secretion. Inhibition of the deiodinases due to elevated serum iodine levels was ruled out (Table 1). The findings are most consistent with a marked decrease in D1 activity including normal rT_3_ concentrations, which might be considered inappropriately low given the elevated T_4_ concentrations, resulting in a high T_4_:rT_3_ ratio (Table 1).

Patient 2 had previously not been diagnosed with hypothyroidism and had a negative TPOA concentration. Unlike in patient 1, the rT_3_ concentration at the upper limit of the reference range was appropriate for the elevated concentration of its substrate T_4_, as indicated by the mid-normal T_4_:rT_3_ ratio (Table 1). It is evidence that the activity of D1 at the 5 position of the inner ring was not impaired. As the enzymatic activities of D1 at the 5 and 5′ positions have been shown to proceed in an equimolar fashion (2), it makes decreased D1 activity an unlikely cause of the low circulating T_3_ levels, suggesting impaired D2 activity as a more likely alternative. She also differed from patient 1 in not having an elevated TSH concentration, albeit that the 1.5 concentration was higher than expected for a T_4_ concentration at the upper limit of the reference range (Table 1). It has long been known that TSH secretion is controlled by a negative feedback mechanism responsive to circulating T_4_ concentrations (1). It is the concentration of T_3_ at the nuclear T_3_ receptors of the thyrotrophs produced by the resident D1 and D2 enzymes as well as the T_3_ from the circulation that ultimately controls TSH secretion (2). Christoffolete *et al*. (13) have demonstrated a unique behaviour of D2 in thyrotrophs in being able to overcome ubiquitination, thereby sustaining net increased T_3_ production at increasing T_4_ concentrations. One might speculate that in patient 2 the postulated decreased D2 activity might be sufficiently ameliorated by its atypical expression in the thyrotrophs to result in no or only minimally elevated TSH levels. An additional possible mechanism may be that reduced T_3_ levels in the hypothalamus caused by decreased D2 activity in the third ventricle tanycytes have reset the TSH response of the thyrotrophs (14). The lack of a markedly elevated TSH concentration in patient 2 may be akin to what has been observed in patients on amiodarone therapy in whom after an initial elevation in TSH concentrations in response to impaired deiodinase activity (11) (12), there is a gradual return to normal or nearly normal levels.

The theoretically possible alternative cause for the low circulating T_3_ levels of a deficiency in T_3_ production by the thyroid gland is not applicable in patient 1 who had Hashimoto's primary hypothyroidism, while in patient 2 the 32% observed reduction from mid-normal levels exceeds the 20% of the total T_3_ contributed by the thyroid (1) (2), and nor would it explain the lack of an increase in T_3_ concentrations as the T_4_ concentration rose in response to increasing the dose of l-T_4_. Furthermore, no such case has been reported. It was not ruled out in either patient that further increases in their T_4_ concentrations by increased doses of l-T_4_ might have resulted in an increase in T_3_ concentrations.

The fact that both patients responded with a decrease in TSH concentrations in response to an increase in circulating T_4_ concentrations indicated an intact negative TSH feedback axis, supporting evidence for the lack of NTIs as a potential cause for the abnormalities found (4) (6) (8). This, together with the lack of clinical evidence that their LT_3_S was secondary to fasting, NTIs or drugs, suggests that the alterations in circulating thyroid hormone levels were not secondary, but might represent primary abnormalities in deiodinase activity. In support of this speculation, Peeters *et al*. (15) have demonstrated polymorphism of deiodinases associated with circulating iodothyronine concentrations and their ratios, while Panicker *et al*. (16) using a similar approach of nucleotide measurement of genes involved in thyroid hormone metabolism found that 26% of patients with primary hypothyroidism on replacement therapy had polymorphism in the *DIO2* gene resulting in a decreased T_4_:T_3_ ratio and clinical manifestations. Considering this frequency in genetic polymorphism, it is not unexpected that patients might be encountered with primary D2 and D1 enzyme abnormalities causing LT_3_S.

## Patient consent

Written consent was obtained from both patients to publish the case report.

## Author contribution statement

The author was the only contributor. The author is a consultant endocrinologist and was and continues to be the physician responsible for the endocrine care of both patients. Both were referred by the family physician.
